# Approaches for the Prediction of Leaf Wetness Duration with Machine Learning

**DOI:** 10.3390/biomimetics6020029

**Published:** 2021-05-14

**Authors:** Martín Solís, Vanessa Rojas-Herrera

**Affiliations:** 1 Tecnológico de Costa Rica, Cartago 159-7050, Costa Rica; 2 Instituto del Café de Costa Rica, Heredia 280-3011, Costa Rica; vrojas@icafe.cr

**Keywords:** Leaf wetness duration, machine learning, coffee leaf

## Abstract

The prediction of leaf wetness duration (LWD) is an issue of interest for disease prevention in coffee plantations, forests, and other crops. This study analyzed different LWD prediction approaches using machine learning and meteorological and temporal variables as the models’ input. The information was collected through meteorological stations placed in coffee plantations in six different regions of Costa Rica, and the leaf wetness duration was measured by sensors installed in the same regions. The best prediction models had a mean absolute error of around 60 min per day. Our results demonstrate that for LWD modeling, it is not convenient to aggregate records at a daily level. The model performance was better when the records were collected at intervals of 15 min instead of 30 min.

## 1. Introduction

The variable of leaf wetness is understood as the presence of water on plant tissues [1]. It is measured as leaf water duration (LWD), which is defined as the time the plant surface shows visible water [2]. This measurement is essential for analyzing and preventing crop, forest, and plant diseases because surface wetness affects fungal and bacterial infection processes [3] and influences the deposition of atmospheric pollutants on leaves [4].

Coffee is one of the most important crops in Costa Rica. It represents 3% of the agricultural sector’s total primary activities [5], and more than 30,000 people directly benefit from these activities [6]. Like any other crop, coffee is faced with many abiotic and biotic factors that influence its maximum potential yield. Diseases are one of the main factors that cause yield losses, and their development is directly associated with weather conditions that vary from one year to the next. In this context, leaf wetness measurement supports the prevention and control strategies that guarantee a successful coffee production each year.

**Figure 1:**
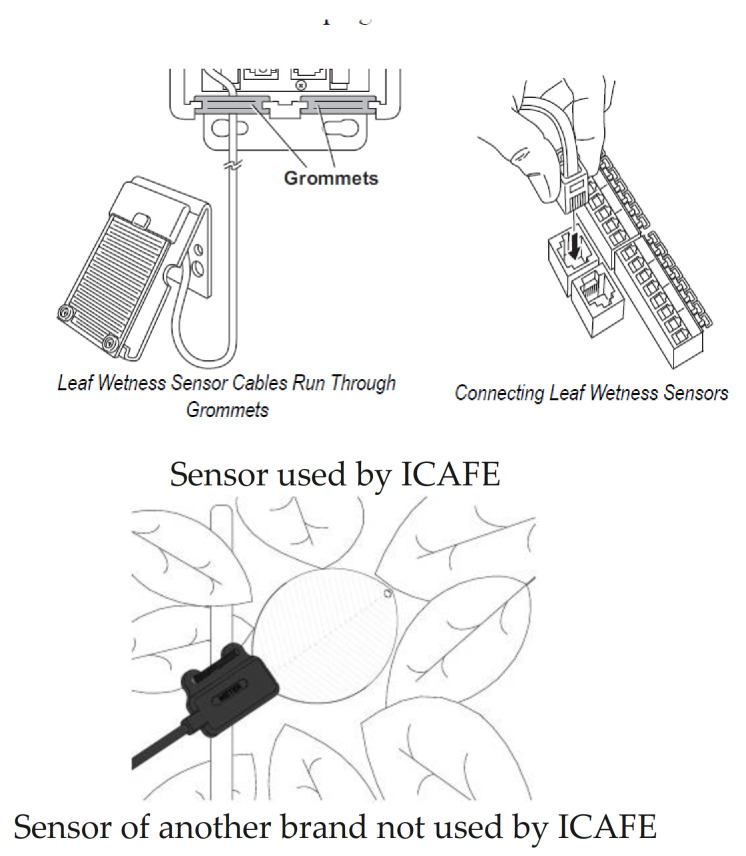
Sensor used by ICAFE and sensor from another brand. Note: Images taken from [8,9].

To this end, the Coffee Institute of Costa Rica (ICAFE) installed sensors to measure leaf water duration (LWD) in regions where there are coffee plantations. Figure 1 shows an example of the kind of sensor used by ICAFE and another kind of sensor not used by ICAFE. The first sensor consists of a sensing grid. The console measures the conductivity across the grid to displays the result as a moisture level. The sensor is mounted against a surface to simulate a typical leaf position. The angle and position of the sensor influence the measurement. Not all kinds of leaf wetness sensors are the same (Figure 1), and the measurements vary from one brand to another [7].

However, use of this procedure to collect LWD has been widely questioned over the years for several reasons: (a) Measurements are affected by the height of sensor installation, angle of deployment, orientation, interaction with surrounding leaves, and timely calibration [10,11]; therefore, two sensors could give different results if the installation is different. (b) There are different types of sensors, and the measurements vary according to type [7]; thus, two sensors of different types in the same place could generate different results. (c) There is no standard protocol for installation of sensors and measurement of LWD, and even the specifications change according to sensor brand [12].

On the other hand, the cost of the sensors can prevent institutions or farmers from buying them. Additionally, there is a cost associated with the monitoring and constant calibration necessary to guarantee data quality. If the sensor is not operated correctly, there may be significant information errors [13,14].

Due to the problems mentioned above, different methods have been proposed to predict LWD based on other variables. First, the physical models: These are accurate but complex, and require many variables that are not always available [15] and are therefore not the best option. Second, empirical and statistical models using meteorological variables as input [10].

The first empirical models predicted LWD based on a threshold of the relative humidity [13,14]. Statistical models using linear and nonlinear regression were then applied to predict LWD. For example, Igarashi et al. [16] used linear regression and meteorological variables obtained from an agrometeorological station to predict leaf wetness percentage at three heights in a soybean (*Glycine max*) canopy in Paraná, Brazil. The majority of their models explained more than 80% of leaf wetness percentage variance. Alcarde et al. [1] developed monthly LWD models by applying nonlinear regression. They achieved correlations of 0.92 and 0.96 between prediction and truth values.

More sophisticated and accurate empirical models have been obtained by applying fuzzy logic and machine-learning models. Kim et al. [10] compared different models’ performances with data from Brazil, Canada, Costa Rica, Italy, and the USA. They concluded that the fuzzy model could be comparable to physical models and has greater spatial portability.

In more recent times, machine-learning models have been used to predict LWD in different countries, showing good performance. The models have been based on artificial neuronal networks [17,18], deep neural networks [19], tree algorithms such as regression tree or CART [20], and ensemble algorithms such as Random Forest [19].

The variables used in the empirical models vary from one study to another, but relative humidity and temperature are frequently included. Other variables used include solar radiation [18], wind speed [10], precipitation [21], hour [19], location [17], and transpiration [2]. Moreover, the period of data collection changes between studies. For example, data have been collected at intervals of 5 min [16], 15 min [20], 30 and 60 min [21], and monthly [1].

Like recent studies, this manuscript proposes a model based on machine learning to predict LWD in coffee leaves, but unlike other studies, this research analyzed alternative approaches that influenced the model’s performance. These approaches were based on the following possibilities: (a) Face the problem as regression or classification; (b) build a model for each region of interest or one general model; (c) train the models with data collected every 30 min or every 15 min. Furthermore, the capability of the model for inter-dataset generalization was analyzed.

## 2. Materials and Methods

### 2.1. Data

**Figure 2:**
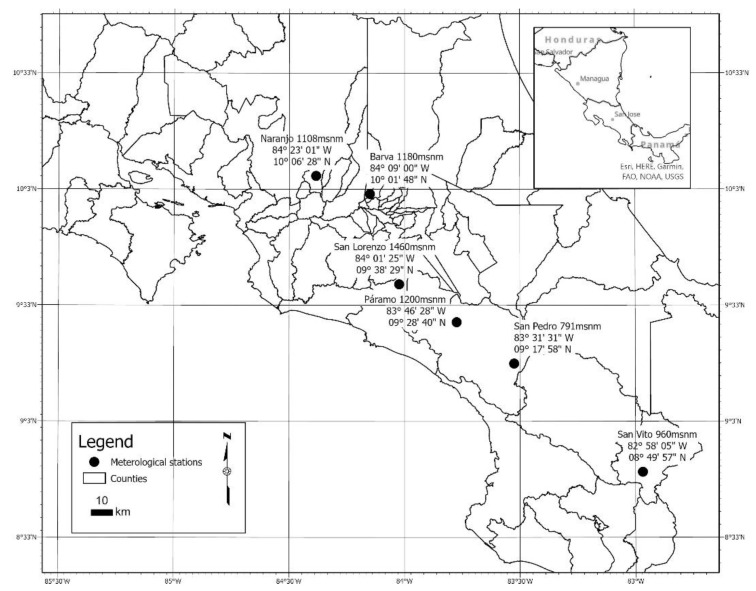
Costa Rica. Regional locations of the meteorological stations and sensors.

The dataset was obtained from meteorological stations and leaf wetness sensors installed by the ICAFE in six regions of Costa Rica. Figure 2 shows the location of each region.

Leaf wetness was measured at each site using a leaf wetness sensor that detected surface moisture. The sensor was an artificial-leaf electrical-resistance type and consisted of a sensing grid, low-voltage bipolar excitation circuit, and conductivity-sensing circuit [21]. The LWD sensors from the six regions were from the same brand and were installed following the manufacturer’s specifications. The sensors were mounted on a vertical pipe deployed at 1.5 m from the ground and facing west at an angle of 45° to simulate a typical leaf position and to permit run-off of excess moisture. Additionally, the sensors were monitored and maintained by ICAFE to guarantee the quality of the information. All the sensors reported leaf wetness level on a point scale from 0 to 15 at given time intervals. For coffee, agronomists generally consider a leaf to be wet if the sensor indicates a value of 1 or higher at a time point.

The input variables were obtained from the meteorological stations. Regions 1, 2, and 3 were calibrated to report values every 15 min, and regions 4, 5, and 6 reported values every 30 min. There were two types of input variables for the machine-learning model: (1) temporal variables of the month (between 1 and 12) and year day (between 1 and 365); (2) the 13 meteorological variables shown in Table 1. In addition, Table 1 shows the descriptive statistics of the meteorological variables and wet leaf percentage for each region. Some variables were similar between regions, but there were no regions with similarities in all variables. For example, regions 1 and 4 had similar temperature, humidity, and solar radiation but different soil moisture; regions 3 and 6 had similar temperature, humidity, and barometric readings but different solar radiation. The wet leaf percentage was similar for stations 1, 4, and 5 with a value around 43%, while in region 2 and 3, high percentages of periods had a wet leaf, with 62% and 57% respectively, and region 6 showed the lower percentage with 38%.

### 2.2. Models and Approaches

XGBoost was used to develop the models for LWD. XGBoost is a scalable derivation of Gradient Boosting Machines and is widely used by data scientists [22] to improve the state of the art in regression and classification problems; therefore, it was also used in this problem. More specifically, it is an ensemble algorithm of trees where each tree considers the error of the previous one. It has an objective function composed of a loss function that measures the difference between predicted and real values, and a regularization part that penalizes the tree’s complexity. A gradient descent algorithm is used to minimize the objective and learn each new tree incorporated into the model. Mathematically, it can be expressed as

(1) $${\hat{y}}_{i}=\overset{k}{\underset{k=1}{\sum }}f_{k}\left(x_{i}\right),\;f_{k}\in f$$

where *k* = number of functions given by the trees, ${\hat{y}}_{i}$ = prediction of instance, $f_{k}$ = function given by a tree, and $f_{k}\left(x_{i}\right)$ = prediction score given by the k-th tree to the *i*-th sample.

The objective function used to defined each tree can be expressed as

(2) $$obj=\sum_{i=1}^{n}l\;\left(y_{i},{\hat{y}}_{i}\right)+\sum_{k=1}^{k}\Omega \left(f_{k}\right),\;\Omega \left(f_{k}\right)=\gamma T+0.5\lambda {\left\|w\right\|}^{2}$$

where *l* = train loss function that measures the distance between real and prediction, Ω = regularization for tree complexity penalization, γ = regularization parameter, *T* = number of leaves on the tree, λ = regularization parameter, and w = score on each leaf.

Each model took a different approach for LWD modeling. These were as follows:

- Daily records for multiple regression models (DMR). One model for each region, using as the output variable the daily LWD in minutes. The input variables were aggregated to a daily level.
- Daily records for one regression model (DOR). One model for the three regions that collected the information every 15 min and one model for the three regions that collected the information every 30 min. Both models used as the output variable the LWD daily in minutes. The input variables were aggregated to a daily level.
- Hourly records for one regression model (HOR). The difference between this model and Model b is that the variables were aggregated by hour instead of by day. To test the model’s performance, the records were aggregated to minutes of daily wetness.
- Natural time records for multiple classification models (NMC). One model for each region, using as the output a dummy variable, where 1 = wet and 0 = not wet, for every 15 or 30 min. When the sensor showed a value greater than 0 at the time interval, the value was converted to 1 because this indicated that the leaf was not completely dry, influencing fungal and bacterial infection processes. To test the model’s performance, the dichotomous prediction was transformed to minutes of wetness during a day. For example, if the prediction was “wet” in an interval of 15 min, it was converted to 15 min of wetness. Finally, the records were aggregated to minutes of daily wetness.
- Natural time records for one classification model (NOC). One model for regions that collected information every 15 min and one model for regions that collected information every 30 min. The output was the dummy variable, where 1 = wet and 0 = not wet, as explained previously.

The first three approaches were treated as a regression case and the last two as a classification case.

### 2.3. Procedure 

#### 2.3.1. Preprocessing and Data Division

Records with missing information (between 4% and 18% of the total days) were deleted. We preferred deletion instead of imputation to avoid the creation of artificial data for many records. After that, the records of the last 1300 days were used for the analysis. The information ranged between January 2016 and September 2020. Finally, the 1300 records were randomly divided into 75% for training and tuning and 25% for validation.

#### 2.3.2. Training 

We applied 10-fold cross-validation with the training dataset (75% of our original dataset) for hyperparameter optimization. Therefore, the training set (75% of our original dataset) was divided into training and testing 10 times for each parameter combination to select the best model. This procedure consisted of taking 9/10 of the sample to calibrate the algorithm with specific parameters and 1/10 to predict the observations. It was replicated 10 times (10 nonoverlapping training and testing sets). At the end of the process, these 10 replications’ predictions were averaged for each parameter combination. Various combinations of parameters were tested, but only the best was chosen, according to the minimization of the loss function, which was the squared mean error for the regression XGBoost and the log loss (binary cross-entropy) for the classification XGBoost. The XGB Boost parameters evaluated were as follows: eta between 0.01 and 0.1, max depth between 6 and 9, min_child_ lambda between 1 and 20.

#### 2.3.3. Validation

Each model’s predictive capacity was evaluated using the validation test. The mean absolute error and root mean absolute error were applied for the evaluation. These metrics were calculated as follows:

(3) $$MAE=\frac{\sum_{i=1}^{n}y_{i}-\hat{y_{i}}}{n}\;RMSE=\sqrt{\frac{\sum_{i=1}^{n}{\left(y_{i}-\hat{y_{i}}\right)}^{2}}{n}}$$

Additionally, for each region, the ANOVA for repeated measures, post hoc multiple paired *t*-test with 5% significance, and Bonferroni correction (for means comparison) were applied to determine whether there was a statistical significance difference between approaches. When the sphericity assumption was violated, we used the Greenhouse–Geisser correction.

## 3. Results

Table 2 shows the mean absolute error for each model and region. The approaches with the best performances in regions 1, 2, 3, 5, and 6 were HOR, NMC, and NOC. Furthermore, there were no significant differences between them according to the post hoc multiple paired *t*-test with 5% significance and Bonferroni correction. In region 4, the best model was NMC, but it had a similar mean absolute error to models HOR and NOC. For each region, the worst models were DMR and DOR. This suggests that aggregation of the records to a daily period deteriorated the model performance. Another relevant finding shown in Table 1 is that the models from regions where the records were collected every 15 min performed better than models from regions where records were collected every 30 min.

There were three approaches with similar performance; however, NMC is less portable because there is a model for each region, while HOR and NOC are based on one general model. The NOC approach is simpler than HOR because it does not require modification of the original variables’ values to an aggregated level. For this reason, we decided to choose NOC to more deeply analyze other elements of LWD prediction.

**Figure 3:**
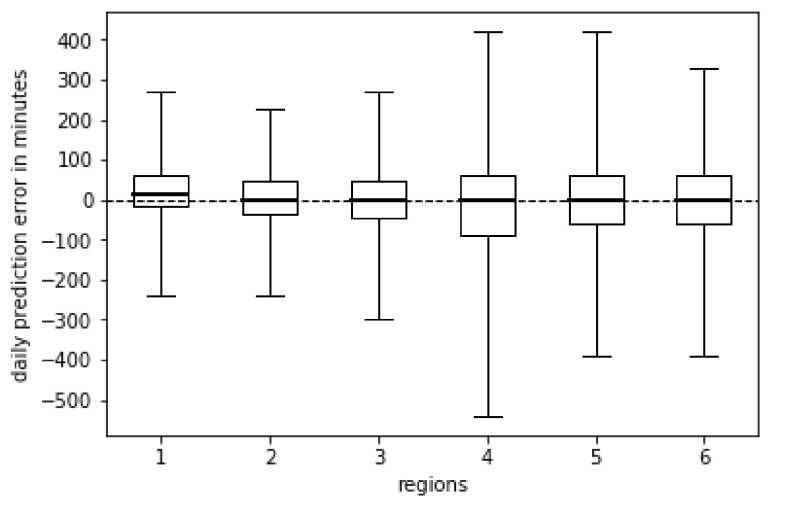
Residual distribution of the NOC approach for each region.

A favorable characteristic of NOC is that it did not widely underestimate or overestimate the real values (Figure 3) since the median was centered or close to zero, and there was not a strong tendency towards positive or negative residuals. It caused a decrease of the error estimation when the LWD was analyzed over several days; for example, the daily mean absolute errors of regions 1, 2, 3, 4, 5, and 6 decreased to 28, 26, 30, 61, 43, and 40 min, respectively, when the LWD was aggregated to periods of seven days.

The model’s capability to make accurate predictions with datasets not used for training is a desirable characteristic. It implies that a general model can be used to make predictions for other regions without the need to retrain the model to incorporate new information. In order to analyze this attribute, each region was excluded from the training process and evaluated with the test dataset. The results showed that the capacity to generalize was low. The mean absolute error went from values close to 60 min to values that exceeded 120 min for the regions excluded with records every 15 min, while in the other regions, there was a greater decrease (Table 3).

**Figure 4:**
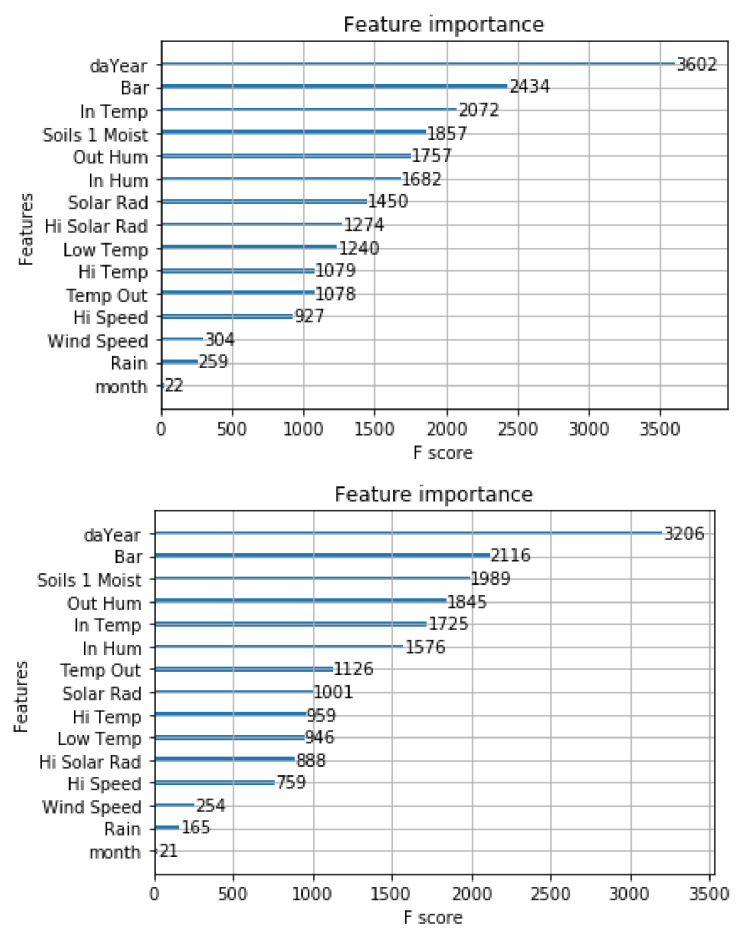
NOC feature importance for records collected each 15 min and records collected each 30 min, respectively.

We measured the relevance of the NOC model variables via the F score, which is the number of times a variable appears in all the trees of the XGBoost model. The main principle of this measurement is to give more importance to the variables used more in the ensemble tree model. Figure 4 shows that the day of the year (daYear) was the most influential variable in both models. It is conceivable that this happened because the variable captured the seasonality of the meteorological conditions that determine the leaf wetness. This finding is interesting because other studies did not use the year day. The least essential variables in both models were month, rain, wind speed, and high speed.

It is relevant to reduce the feature space because fewer features imply fewer sensors and a lower cost to generate the model’s input. We developed three models with fewer variables using the NOC approach. These models were compared with the NOC model that included all the variables. First, in NOC_1, we excluded variables with high correlation to others, namely high temperature, low temperature, and high solar radiation, and four variables that showed less importance in the XGBoost models considering the F score, namely high speed, rain, wind speed, and month. Second, in NOC_2, we excluded the same variables as NOC_1 and the variable in_humidity because it showed the lowest importance in NOC_1 considering the F score. Finally, in NOC_3, we excluded solar radiation because it showed the lowest importance in NOC_2 models considering the F score.

Table 4 shows that in NOC_1, the mean absolute error increased, but not overmuch in most regions. Even in regions 4 and 5, there was no significant difference compared to the model with all variables, suggesting that it can be used as a simple alternative. The exception was region 6, where the MAE increase was close to 20 min. For NOC_2 and NOC_3, the MAE increased again, mainly in the regions where data were collected every 30 min.

We analyzed whether it was better to train the model using more current data rather than including information from years distant from the present. Therefore, the MAE for the dataset of 2019 and 2020 was calculated for the NOC model trained with all the years; the NOC model trained with 2018, 2019, and 2020; and the NOC model trained with 2019 and 2020. This information is shown in Table 5. The results showed that the MAE was very similar in five regions. The exception was region 4, where the MAE decreased considerably when the model was built using more recent periods. This finding suggests that it is necessary to pay attention to the use of periods distant from the present to train the model. For some regions, it could be required to update the model more frequently. It would be relevant to explore this issue by analyzing the behavior of several areas with similar weather conditions.

## 4. Conclusions

The performance of the models appears suitable when compared with other studies. Our best model had a mean absolute error of around 60 min, while Kim et al. [10] reported a mean absolute error of 150 min, Park et al. [19] reported a mean absolute error between 90 and 174 min with their best models, and Jian et al. [17] reported mean absolute errors of 109 min and [2] of 90 and 111 min. The previous comparisons must be made with care because it is clear that neither the data used for training and testing nor the variables were the same, but they may serve as references.

Our results demonstrate that for the LWD modeling, it was not suitable to aggregate the records at a daily level because of the models’ worsened performance. Two possible alternatives are (a) to aggregate the record by hour if they were collected in a shorter period and model the leaf wetness using a regression algorithm; (b) to use the records in the period in which they were collected and model leaf wetness as a dichotomous response variable using a classification algorithm. In addition, the results indicate that it is better to collect the data in periods of 15 min than in periods of 30 min. Other studies about LWD prediction have not questioned whether the period of analysis influences the models’ performance, and there is therefore frequent variation between studies in the period used.

The previous results are congruent and suggest that records should be collected and modeled in shorter periods to get the most accurate possible models. A possible hypothesis is that when the interval of data collection and analysis is extended, we lose the continuous-time variability from input variables such as temperature, humidity, solar radiation, and the dependent variable. However, to prove this hypothesis it is necessary to collect data from different levels in the same region, or at least to have a broad sample of regions with records collected over diverse periods.

The models did not demonstrate an acceptable ability to predict accurately in regions not included in the training process. Future research should focus on the generalization of the models to make predictions in different regions without the need to collect new information for model retraining. For this task, it is crucial to collect data with a wide diversity of meteorological conditions and regions. Furthermore, the wetting sensors must have a uniform installation and be of the same brand, since these factors alter the measurements [7].

Another idea to improve the models’ performance is the inclusion of lagging values of the input variables, considering that the meteorological condition of period t could influence the leaf wetness in t + 1. To execute this idea, it will be relevant to have datasets without missing values.

## Figures and Tables

**Table 1:** Mean and standard deviation of the variables.

Variables	Unit	1. Barva		2. San Vito		3. San Lor..		4. Naranjo		5. San Ped..		6. Páramo	
		m	std	m	std	m	std	m	std	m	std	m	std
Temp. out_station	°C	21	4	23	3	19	3	22	4	23	3	20	3
High temperature	°C	21	4	23	3	19	3	22	4	23	3	20	3
Low temperature	°C	21	4	23	3	18	3	22	4	23	3	20	3
Temp. in station	°C	24	2	27	4	24	2	26	1	26	3	24	5
Humidity out_station	%	80	14	89	10	86	14	77	13	85	8	91	8
Humidity in_station	%	55	9	60	9	64	7	58	8	65	8	65	9
Solar radiation	W/m²	193	294	172	272	196	298	189	278	141	235	145	233
High solar rad	W/m²	228	338	218	337	233	346	252	355	208	328	207	323
Wind speed	km/h	1	2	1	2	2	4	2	2	1	1	2	3
High speed	km/h	8	8	4	5	7	9	7	7	5	5	7	6
Barometer	hPa	782	1	757	2	758	2	755	37	741	27	760	1
Rain	mm	0.1	0.6	0.1	0.7	0.1	0.4	0.1	0.8	0.2	1.4	0.2	1.0
Soil moisture	cB	193	20	120	71	67	69	116	69	7	15	37	50
Wet leaf (%)	%	0.43	0.49	0.62	0.49	0.57	0.50	0.44	0.50	0.43	0.49	0.38	0.49

Note: m = mean, std = standard deviation. Temp. out_station = Average temperature outside the meteorological station within a given time interval (15 or 30 min); High temperature = Maximum temperature outside the meteorological station within a given time interval; Low temperature = Minimum temperature outside the meteorological station within a given time interval; Temp. in station = Average temperature inside the meteorological station within a given time interval; Humidity out_station = Average humidity outside the meteorological station within a given time interval; Humidity in_station = Average humidity inside the meteorological station within a given time interval; Solar radiation = Average solar radiation within a given time interval; High solar rad = Maximum solar radiation within a given time interval; Wind speed = Average wind speed within a given time interval; High speed = Maximum high speed within a given time interval; Barometer = Average air pressure within a given time interval; Rain = Rain within a given time interval; Soil moisture = Soil moisture within a given time interval; Wet leaf = Percentage of time intervals where the leaf wetness threshold was greater than zero.

**Table 2:** Daily mean absolute error and root mean squared error in minutes, according to regions and approaches.

Station	DMR		DOR		HOR		NMC		NOC	
	MAE	RMSE	MAE	RMSE	MAE	RMSE	MAE	RMSE	MAE	RMSE
15_min										
1	96 ^b^	139	96 ^b^	135	65 ^a^	91	65 ^a^	96	62 ^a^	90
2	82 ^b^	105	80 ^b^	106	54 ^a^	72	53 ^a^	75	57 ^a^	79
3	92 ^b^	123	91 ^b^	123	64 ^a^	98	64 ^a^	92	65 ^a^	93
30_min										
4	123 ^c^	175	124 ^c^	181	102 ^b^	140	96 ^a^	136	99 ^b^	146
5	125 ^b^	161	126 ^b^	162	95 ^a^	128	86 ^a^	124	88 ^a^	129
6	113 ^b^	145	119 ^b^	150	83 ^a^	107	81 ^a^	113	84 ^a^	111

Note: a = the smallest averages between approaches, according to the post hoc multiple paired *t*-test at 5% significance and Bonferroni correction; b = the second smallest averages; c = the third smallest averages.

**Table 3:** Daily mean absolute error in minutes with excluded regions in training.

Test Sample	Train Sample		
	Without 1	Without 2	Without 3
1	127 *	61	63
2	54	135 *	55
3	66	64	168 *
	**Without 4**	**Without 5**	**Without 6**
4	351 *	97	96
5	87	387 *	86
6	83	85	363 *

* *p* < 0.05, difference between excluding and not excluding, after applying paired *t*-test for comparison of means.

**Table 4:** Daily mean absolute error in minutes for NOC models with data reduction.

Region	NOC_All Variables	NOC_1	NOC_2	NOC_3
15_min				
1	63	67 *	71 *	69 *
2	55	61 *	63 *	68 *
3	66	72 *	75 *	79 *
30_min				
4	99	104	123 *	132 *
5	88	94	131 *	137 *
6	84	103 *	101 *	102 *

NOC_all variables = model with all the variables, NOC_1 = excluded high temperature, low temperature, high speed, high solar radiation, rain, wind speed, and month; NOC_2 = excluded the same features as NOC_1 and humidity; NOC_3 = excluded the same features as NOC_2 and solar radiation. * *p* < 0.05, the difference between NOC reduced and NOC original after applying paired *t*-test for comparison of means.

**Table 5:** Daily mean absolute error in minutes for the test sample of 2019 and 2020, using NOC models trained with different records.

Region	NOC_All	NOC_>2017	NOC_>2018
15_min			
1	57	56	55
2	45	46	43
3	65	64	64
30_min			
4	131	117	100
5	76	76	80
6	90	94	89

NOC_all = NOC model trained with all records; NOC_>2017 = NOC model trained with the years 2018, 2019, and 2020; NOC_>2018 = NOC model trained with the years 2019 and 2020.

## Data Availability

The datasets are available on request from the corresponding author.
